# Construct a clinical prediction model of cumulative live birth rate for IVF/ICSI in female patients of different ages

**DOI:** 10.1530/RAF-25-0062

**Published:** 2025-10-08

**Authors:** Chunya Ye, Panhavolak Chhoon, Hedong Lu, Min Li, Xiya Jiang, Lanxin Xie, Dongmei Ji, Zhiguo Zhang, Yunxia Cao, Weiwei Zou

**Affiliations:** ^1^ Reproductive Medicine Center, Department of Obstetrics and Gynecology, The First Affiliated Hospital of Anhui Medical University, Hefei, China; ^2^ NHC Key Laboratory of Study on Abnormal Gametes and Reproductive Tract (Anhui Medical University), Hefei, China; ^3^ Key Laboratory of Population Health Across Life Cycle (Anhui Medical University) Ministry of Education of the People’s Republic of China, Hefei, Anhui, China

**Keywords:** infertile women, IVF/ICSI, number of retrieved eggs, cumulative live birth rate, clinical prediction model

## Abstract

**Abstract:**

In order to determine the number of eggs to be retrieved to maximize live birth outcomes, this study aimed to develop a clinical prediction model that would identify important predictors of cumulative live birth rate after intracytoplasmic sperm injection or *in vitro* fertilization in women of different ages. A total of 374 infertile women undergoing assisted reproductive treatment at the First Affiliated Hospital of Anhui Medical University between December 2020 and December 2023 were included and categorized into three age groups: <35 years, 35–39 years, and 40 years or older. Clinical data, laboratory results, ovulation induction parameters, and pregnancy outcomes were examined. Least absolute shrinkage and selection operator regression was used for predictive modeling, and linear regression equations were used to measure the correlation between the probability of a live birth and the quantity of retrieved eggs. The number of metaphase II eggs and high-score blastocysts were found to be the most predictive factors in women under 35, with live birth probabilities of 99% after 15 eggs were recovered. The most predictive factors among women aged 35–39 were the number of follicles and metaphase II eggs. The live birth probability was 90% when 20 eggs were retrieved. A live birth was predicted by the quantity of retrieved oocytes only for women aged 40 years or older; retrieving 14 eggs resulted in a 50% chance of giving birth. The proposed model provides age-specific recommendations for egg retrieval to improve reproductive outcomes and reduce the risk of overstimulation.

**Lay summary:**

*In vitro* fertilization (IVF) and intracytoplasmic sperm injection (ICSI) are important medical options that help couples with infertility have children. However, many women now delay having children until later in life, which can make it more difficult to become pregnant even with these treatments. As women age, both the number and quality of their eggs decline, and their chances of a successful pregnancy decrease. This study aimed to help doctors better predict the chances of a successful birth from IVF or ICSI treatments by creating a clinical prediction model. In this study, we selected 374 women of different age groups who underwent IVF/ICSI and analyzed how different factors, such as age and the number of retrieved eggs, affected the chances of giving birth. For example, in women under 35 years old, retrieving ten eggs results in a live birth probability of over 50%, while retrieving 15 and 20 eggs increases that chance to 99% and nearly 100%, respectively. In women aged 35–39, the live birth rates are approximately 60–70% with 15 eggs, 90% with 20 eggs, and over 95% with 25 eggs. For women aged 40 or older, retrieving 14 eggs gives about a 50% chance of live birth. This model helps doctors personalize treatment plans based on a woman’s age, improving the chance of success while minimizing risks such as overstimulation of the ovaries. The study found that age plays a major role; generally, younger women need fewer eggs to achieve a high chance of giving birth.

## Introduction

The development of assisted reproductive technology (ART), especially *in vitro* fertilization-embryo transfer (IVF) and intracytoplasmic sperm injection (ICSI), has enabled infertile couples around the world to find a way to have children and hope (Van Voorhis 2006). However, due to the continuous increase in the number of older pregnant women, research on the reproductive problems of older women has also deepened worldwide (Garrido *et al.* 2011). International academics have published a series of research results on the physiological and technical difficulties faced by older women in the process of applying ART (Carmona-Gutierrez *et al.* 2016). These research results not only provide older women with more opportunities for childbearing but also promote scientific progress in the field of reproductive medicine. Female age is one of the most important factors affecting the likelihood of pregnancy in ART programs (Liu & Case 2017). With the development of modern technology, the education and employment rates of women have gradually increased, and the age of marriage has also gradually increased. The age of couples receiving IVF/ICSI-assisted pregnancy is gradually increasing worldwide. Among women who have received IVF in the past decade, the proportion of those aged 40 and above has increased the fastest (Gleicher *et al.* 2016). At the same time, women also have more social pressure and family responsibilities. The mental and psychological problems caused by life pressure also have an impact on women’s fertility (El Kissi *et al.* 2014). The cumulative live birth rate is an important indicator for evaluating the treatment effectiveness of ART, such as IVF/ICSI (*in vitro* fertilization/intracytoplasmic sperm injection). Identifying the key indicators affecting cumulative live birth rate can help guide the controlled ovarian stimulation (COS) process in IVF/ICSI. The basic clinical data of the patient and the important indicators in the IVF/ICSI process may be the key factors affecting the cumulative live birth rate. Several studies have shown that there is a strong correlation between the number of retrieved oocytes and the cumulative live birth rate, which is an important factor affecting the clinical outcome of IVF/ICSI (Le *et al.* 2021). As the number of retrieved oocytes increased, the cumulative live birth rate of the patients gradually increased (Gao *et al.* 2021). Therefore, the number of retrieved oocytes can be used as one of the indicators to predict the cumulative live birth rate. Age is an important index affecting the number of retrieved oocytes. The quality and quantity of oocytes decrease significantly, and the ovarian reserve function, response to exogenous gonadotropin, embryo implantation rate, and clinical pregnancy rate all decrease in women over 35 years old. The incidence and abortion rate of genetically abnormal fetuses have increased significantly (Ziebe *et al.* 2001, Liu *et al.* 2022).

In recent years, clinical prediction models have been widely used in medical research and practice. With the help of clinical prediction models, doctors and patients can better make joint decisions and estimate the probability of developing a certain disease or the probability of having a certain future outcome by using a multi-factor model (Dong *et al.* 2021). This study aims to calculate the best predictor of cumulative live birth rate for patients of different ages, construct a nomogram, and then combine the linear regression equation for the optimal number of retrieved oocytes to achieve the maximum probability of live birth, so as to provide guidance for the clinical process of COS.

## Materials and methods

In this study, 374 patients who received IVF/ICSI treatment in the First Affiliated Hospital of Anhui Medical University from December 2020 to December 2023 were selected. The basic clinical data, ovulation induction data, IVF/ICSI laboratory results, and clinical outcomes of these patients were collected. All patients received IVF/ICSI treatment, and the obtained embryos were frozen, and then frozen/thawed embryos were transferred. Using live birth as the prognostic criterion, a clinical prediction model was constructed to screen for the best predictors of cumulative live birth rate in women in different age groups (<35 years old, 35 ≤ age <40 years old, and ≥40 years old), and a nomogram was constructed to obtain the optimal number of retrieved eggs to achieve the maximum probability of live birth by linear regression equation.

### Study subjects

#### Inclusion criteria

- Infertility patients.
- Infertility factors excluding uterine factors and male factors.
- Completed a single IVF/ICSI cycle; with complete clinical data, complete variable information, and with pregnancy outcome clearly known.
- The embryos obtained by the patient were frozen, followed by freeze-thawed embryo transfer.

#### Exclusion criteria

- The infertility factors were uterine factors such as endometrial polyps, submucosal fibroids, or endometriosis.
- Lack of important clinical data.
- Pregnancy outcome was unclear and loss to follow-up.
- Fresh cycle of embryo transfer.

### Ethics approval

This study was approved by the Institutional Review Board (IRB) of The First Affiliated Hospital of Anhui Medical University, Anhui, China. Approval Number: JP 2024-2024-12-30. The protocols used in the study were approved by The First Affiliated Hospital of Anhui Medical University. A total of 374 infertile women undergoing IVF/ICSI at The First Affiliated Hospital of Anhui Medical University (Dec 2020–Dec 2023) provided informed consent forms according to institutional guidelines, and the study was approved by the Institutional Review Board (IRB) protocols of The First Affiliated Hospital of Anhui Medical University.

### Study methods

After assessing the basic clinical data of female patients, the protocol of COS was developed, including but not limited to the use of ovulation-stimulating drugs such as letrozole, clomiphene, or urinary gonadotropin, as well as ovulation-stimulating protocols such as antagonist, microsimulation, and long luteal phase protocols. On the day of oocyte retrieval for the female patients, the male collected a semen sample. For patients undergoing IVF treatment, the cumulus–oocyte complex (COC) and sperm were placed into the fertilization medium (Sage, USA) and granulated 4–5 h later to observe the discharge of the second polar body. In patients treated with ICSI, cumulus cells were removed (oocyte denudation) before ICSI insemination. The fertilized oocytes were transferred to a plate containing cleavage medium (Sage, USA) and cultured until day 3 after fertilization, then put into blastocyst medium (Sage, USA) until day 5 or 6. The blastocysts obtained by the patients were frozen on day 5 or 6 after fertilization and then stored in liquid nitrogen at −196°C. In a frozen-thawed embryo transfer cycle, the endometrium was prepared by artificial cycles or natural cycles. On day 5 of ovulation or day 5 of progesterone injection, the embryos were thawed and transferred under abdominal ultrasound guidance. At 30 days after the embryo transfer, if the pregnancy sac, germ, and heartbeat were observed in the uterine cavity, the clinical pregnancy was confirmed, and the patient continued to receive luteal support until 65 days after transplantation. The patients were followed up during pregnancy and postpartum, and the related information was recorded. The calculation of cumulative live birth rate in this study was calculated as the cumulative live birth rate for a single oocyte retrieval cycle according to the 2018 Chinese expert consensus (Reproductive Medicine Committee of the Chinese Medical Doctor Association 2018).

### Statistical analysis

In this study, data conforming to normal distribution were represented by mean ± standard deviation, while data not conforming to normal distribution were represented by median and interquartile range (IQR). The calculation of cumulative live birth rate in this study refers to the 2018 Chinese expert consensus in the training cohort. Potential prognostic factors were screened using Least Absolute Shrinkage and Selection Operator (LASSO) regression, The LASSO regression was performed using the glmnet package in R software (version 4.4.1). A ten-fold cross-validation method was employed to determine the optimal penalty parameter lambda that minimized the cross-validation mean squared error. Variables with non-zero coefficients were selected as the most predictive features. A nomogram was constructed using the rms package in R, based on the predictors identified through LASSO regression. The nomogram graphically represented the contribution of each variable to the cumulative live birth rate, with point values assigned proportionally to their regression coefficients. Internal validation of the model was conducted through 1,000 bootstrap resampling iterations. The performance of the prediction model was evaluated using the receiver operating characteristic (ROC) curve, area under the curve (AUC), Harrell’s C-index, and calibration plots, comparing predicted versus observed probabilities. A *P*-value of less than 0.5 was considered statistically significant.

## Results

### Basic clinical data, ovulation induction data and laboratory data of the three groups of patients

In this study, there were a total of 96 female patients under the age of 35, of whom 63 obtained live births and 33 obtained non-live births after a single egg retrieval cycle. Live births were defined as 1 and non-live births as 0. The basic clinical data, ovulation induction data, and IVF/ICSI laboratory results were collected in this study, including age, infertility years, body mass index (BMI), basic hormone level, gonadotropins (Gn), Gn days used, number of follicles under ultrasound, number of retrieved eggs, number of metaphase II (MII) eggs, number of blastocysts, number of transferable embryos, and number of high-score blastocysts. After statistical analysis, statistically significant indicators included the number of retrieved eggs, the number of MII eggs, the number of blastocysts, the number of transferable embryos, and the number of high-score blastocysts (*P* < 0.001) (Table 1).

**Table 1:** General situation of infertile patients less than 35 years old with live births (1) and non-live births (0). Data are presented as mean (SD).

Variables	0 (*n* = 33)	1 (*n* = 63)	*P*
Age	28.79 (3.63)	28.63 (3.19)	0.832
Infertility-years	2.90 (2.55)	2.97 (2.19)	0.892
BMI	22.52 (3.05)	21.79 (2.74)	0.238
FSH	7.95 (2.39)	7.41 (2.09)	0.253
Estradiol	195.75 (153.86)	175.47 (97.16)	0.432
Progesterone	2.21 (1.07)	2.30 (1.40)	0.761
Luteinizing hormone	4.79 (2.96)	5.50 (3.21)	0.298
Use of Gn	2,389.58 (790.18)	2,075.24 (647.78)	0.039
Usage days of Gn	10.39 (1.71)	10.87 (1.86)	0.222
Number of follicles	10.58 (5.40)	13.76 (5.04)	0.005
Number of retrieved oocytes	11.73 (6.62)	17.97 (7.68)	<0.001
MII	7.45 (5.13)	13.71 (5.46)	<0.001
Number of transferable embryos	3.48 (3.62)	6.90 (3.64)	<0.001
Cultivate to blastocyst count	3.27 (3.72)	6.97 (3.72)	<0.001
Number of high-score blastocyst	1.76 (2.46)	5.02 (3.25)	<0.001

FSH, follicle-stimulating hormone; BMI, body mass index; Gn, gonadotropins.

There were 189 female patients aged 35 years and less than 40 years, of which 96 were live births and 93 were non-live births. After statistical analysis, statistically significant indicators included age, the number of follicles, the number of retrieved eggs, the number of MII eggs, the number of blastocysts, the number of transferable embryos, and the number of high-score blastocysts (*P* < 0.001) (Table 2).

**Table 2:** General situations of infertile patients aged 35 years and above but less than 40 years old with live births (1) and non-live births (0). Data are presented as mean (SD).

Variables	0 (*n* = 93)	1 (*n* = 96)	*P*
Age	37.30 (1.49)	36.46 (1.40)	<0.001
Infertility years	3.56 (3.30)	3.85 (4.12)	0.597
BMI	23.73 (3.18)	22.80 (2.88)	0.037
FSH	9.13 (5.26)	7.62 (2.63)	0.013
Estradiol	482.64 (1,150.62)	382.25 (1,674.90)	0.635
Progesterone	3.99 (7.93)	4.94 (14.37)	0.588
Luteinizing hormone	4.47 (2.11)	5.03 (4.04)	0.243
Use of Gn	2,855.51 (1,059.87)	2,459.27 (897.87)	0.006
Usage days of Gn	10.73 (2.28)	10.35 (2.03)	0.231
Number of follicles	6.52 (3.99)	10.13 (5.85)	<0.001
Number of retrieved oocytes	7.78 (5.65)	12.55 (6.97)	<0.001
MII	5.83 (3.73)	9.21 (5.13)	<0.001
Number of transferable embryos	2.65 (2.26)	4.15 (2.57)	<0.001
Cultivate to blastocyst count	2.62 (2.28)	4.11 (2.55)	<0.001
Number of high-score blastocysts	1.99 (1.92)	3.29 (2.36)	<0.001

FSH, follicle-stimulating hormone; BMI, body mass index; Gn, gonadotropins.

There were 71 female patients over 40 years of age, including 14 live births and 57 non-live births. After statistical analysis, statistically significant indicators included the number of follicles, the number of retrieved eggs, and the number of MII eggs (*P* < 0.001) (Table 3).

**Table 3:** General situation of infertile patients over 40 years old with live births (1) and non-live births (0). Data are presented as mean (SD).

Variables	0 (*n* = 57)	1 (*n* = 14)	*P*
*n*	57	14	
Age	42.32 (2.16)	42.21 (2.83)	0.883
Infertility-years	4.57 (5.59)	2.76 (2.86)	0.245
BMI	23.80 (3.06)	22.96 (2.72)	0.351
FSH	9.33 (3.76)	7.92 (1.48)	0.175
Estradiol	576.81 (1,207.57)	451.42 (661.88)	0.71
Progesterone	2.79 (2.92)	11.25 (23.60)	0.009
Luteinizing hormone	4.21 (2.88)	5.05 (3.16)	0.257
Use of Gn	2,626.09 (1,109.22)	2,711.61 (583.06)	0.782
Usage days of Gn	9.68 (2.28)	10.57 (1.60)	0.174
Number of follicles	5.19 (3.12)	8.64 (4.78)	0.001
Number of retrieved oocytes	5.47 (3.42)	11.79 (7.63)	<0.001
MII	4.35 (2.85)	8.79 (5.29)	<0.001
Number of transferable embryos	1.86 (1.52)	3.14 (1.79)	0.008
Number of blastocysts	1.82 (1.53)	3.14 (1.79)	0.007
Number of high-score blastocysts	1.00 (1.43)	1.64 (1.15)	0.123

FSH, follicle-stimulating hormone; BMI, body mass index; Gn, gonadotropins

### Correlation analysis and screening of predictors

**Figure 1:**
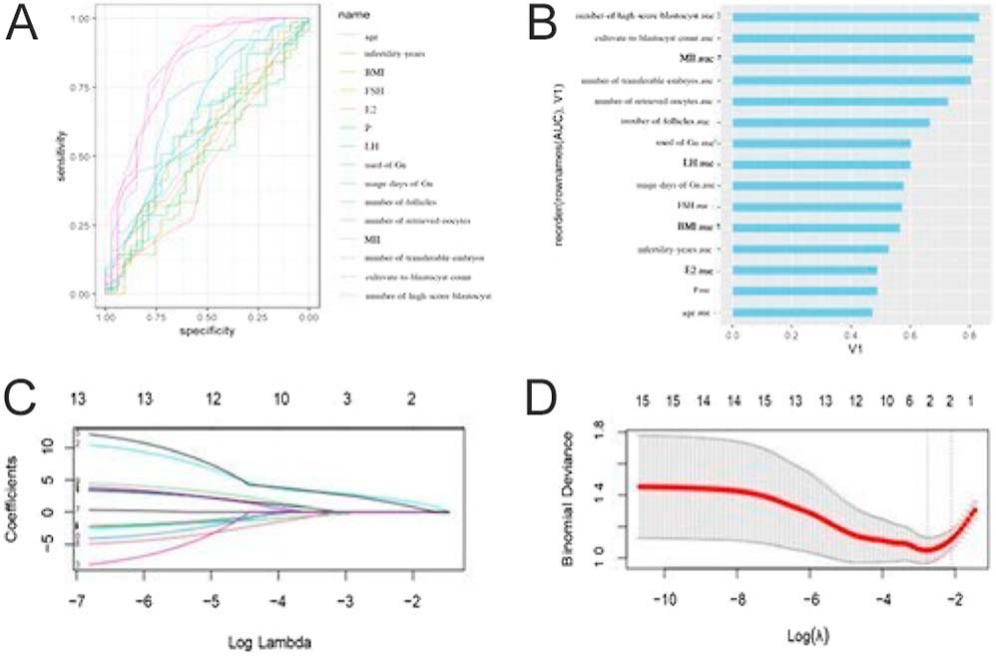
Correlation analysis and screening of predictive factors in infertile female patients aged < 35 years. (A and B) In female patients < 35 years old, the AUC values of the number of follicles under ultrasound, number of retrieved oocytes, number of transferable embryos, number of metaphase II (MII) oocytes, and number of blastocysts were 0.67, 0.73, 0.80, 0.81, and 0.81, respectively. (C) LASSO regression coefficient path diagram. As log λ increases, the regression (penalty) coefficient is eventually compressed to 0, screening out potential predictors. (D) The most suitable predictor is screened by the ten-fold cross-validation method in LASSO regression analysis. When the minimum mean square error (λ-min) is selected, two variables are retained (the position corresponding to the dotted line on the left), and when the distance from the minimum mean square error by 1 standard error (λ1-se) is selected, two variables are retained (the position corresponding to the dotted line on the right).

A live birth was used as a positive indicator. Age, infertility years, BMI, basic hormone level, gonadotropins (Gn), Gn days used, number of follicles under ultrasound, number of retrieved oocytes, number of metaphase II (MII) oocytes, number of blastocysts, number of transferable embryos, and number of high-score blastocysts were used as evaluation indexes. The AUC was used to represent the area under the ROC curve. In female patients <35 years old, the AUC values of the number of follicles under ultrasound, number of retrieved oocytes, number of transferable embryos, number of metaphase II (MII) oocytes, number of blastocysts, and number of high-score blastocysts were 0.67, 0.73, 0.81, 0.81, 0.81, and 0.83, respectively (Fig. 1A and B). LASSO regression selected the number of high-score blastocysts and the number of metaphase II (MII) oocytes as predictors (Fig. 1C and D).

**Figure 2:**
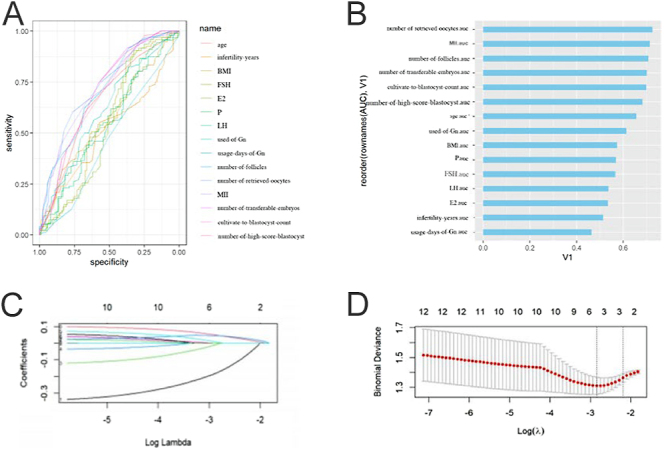
Correlation analysis and screening of predictive factors in infertile female patients aged 35 years and less than 40 years. (A and B) In female patients aged 35 years and less than 40 years, the AUC values of number of blastocysts, number of transferable embryos, number of follicles under ultrasound, number of metaphase II (MII) oocytes were 0.70, 0.70, 0.71, 0.73, 0.71, and 0.69, respectively. (C) LASSO regression coefficient path diagram. As log λ increases, the regression (penalty) coefficient is eventually compressed to 0, screening out potential predictors. (D) The most suitable predictor is screened by the ten-fold cross-validation method in LASSO regression analysis. When the minimum mean square error (λ-min) is selected, three variables are retained (the position corresponding to the dotted line on the left), and when the distance from the minimum mean square error by 1 standard error (λ1-se) is selected, three variables are retained (the position corresponding to the dotted line on the right).

In female patients aged 35 years and less than 40 years, the AUC values of the number of blastocysts, number of transferable embryos, number of follicles under ultrasound, number of metaphase II (MII) eggs, and number of high-score blastocysts were 0.70, 0.70, 0.71, 0.73, 0.71, and 0.69, respectively (Fig. 2A and B). LASSO regression selected the number of follicles under ultrasound and the number of metaphase II (MII) oocytes as predictors (Fig. 2C and D).

**Figure 3:**
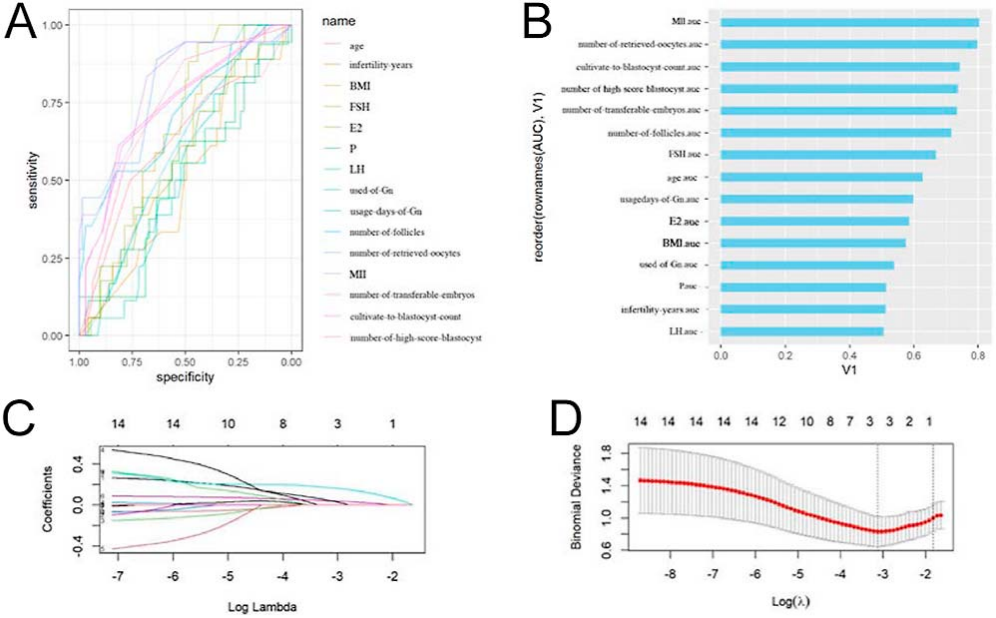
Correlation analysis and screening of predictive factors in infertile female patients over 40 years. (A and B) In female patients over 40 years of age, the AUC values of Age, basic hormone level, number of follicles under ultrasound, number of transplantable embryos, number of blastocysts, number of high-score blastocysts, number of metaphase II (MII) oocytes, and number of retrieved oocytes were 0.63, 0.67, 0.72, 0.73, 0.74, 0.74,0.80, and 0.80, respectively. (C) LASSO regression coefficient path diagram. As log λ increases, the regression (penalty) coefficient is eventually compressed to 0, screening out potential predictors. (D) The most suitable predictor is screened by the ten-fold cross-validation method in LASSO regression analysis. When the minimum mean square error (λ-min) is selected, three variables are retained (the position corresponding to the dotted line on the left), and when the distance from the minimum mean square error by 1 standard error (λ1-se) is selected, one variable is retained (the position corresponding to the dotted line on the right).

In female patients over 40 years of age, the AUC values of age, basic hormone level, number of follicles under ultrasound, number of transplantable embryos, number of blastocysts, number of high-score blastocysts, number of metaphase II (MII) oocytes, and number of retrieved oocytes were 0.63, 0.67, 0.72, 0.73, 0.74, 0.80, and 0.80, respectively (Fig. 3A and B). LASSO regression selected the number of retrieved oocytes as the predictor (Fig. 3C and D).

### Construction of the nomogram and the linear regression equation

**Figure 4:**
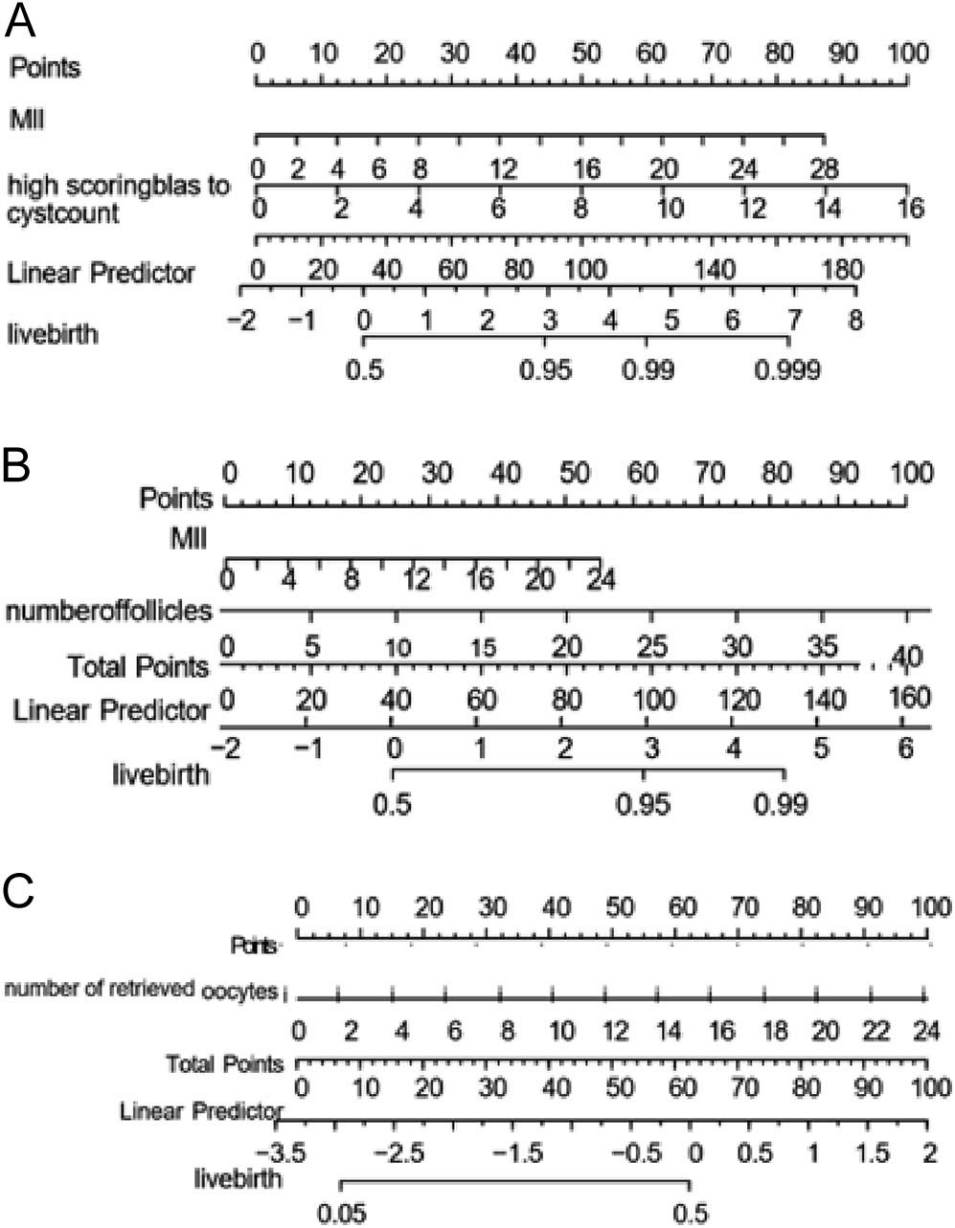
A nomogram for infertile female patients (Prediction Age < 35 years, 35 years ≤ Age < 40 years, Age > 40 years). (A) When the patient is < 35 years old and the number of retrieved oocytes is 10, 15, and 20, the probability of live birth is 50%+, 99%, and 99.9%, respectively. (B) When the patient is 35 ≤ age < 40 years old and the number of retrieved oocytes is 15, 20, and 25, the probability of live birth is 60% – 70%, 90%, and 95%+, respectively. (C) When the patient is > 40 years old and the number of retrieved oocytes is 14, the probability of live birth is 50%.

In female patients <35 years old, the predictors of LASSO screening were the number of high-score blastocysts and the number of metaphase II (MII) eggs. Then a nomogram was constructed and combined with the linear regression equation (the number of metaphase II (MII) eggs = 0.679 × the number of retrieved eggs +0.819 (*P* < 0.001); the number of high-score blastocysts = 0.370 × the number of metaphase II (MII) eggs −0.379 (*P* < 0.001). It can be calculated that the number of retrieved eggs is 10, 15, and 20; the probability of live birth is 50%+, 99, and 99.9%, respectively (Fig. 4A).

In female patients aged 35 years and less than 40 years, the predictors of LASSO screening were the number of metaphase II (MII) eggs and the number of follicles under ultrasound. Then a nomogram was constructed and combined with the linear regression equation MII = 0.645 × the number of retrieved eggs +0.825 (*P* < 0.001); number of follicles under ultrasound = 0.610 × the number of retrieved eggs +2.101 (*P* < 0.001). It can be calculated that when * the number of retrieved eggs is 15, 20, and 25, the probability of live birth is 60%–70, 90, and 95%+, respectively (Fig. 4B).

In female patients over 40 years of age, the predictor of LASSO screening was the number of retrieved eggs. When the number of retrieved eggs was 14, the probability of live birth was 50% (Fig. 4C).

## Discussion

Studies (Fatemi *et al.* 2012, Devesa *et al.* 2018) have confirmed that the cumulative live birth rate of IVF/ICSI increases with the number of retrieved oocytes during the oocyte retrieval cycle, but the relationship between the number of retrieved oocytes and pregnancy outcome has not been clear. A previous study (Sunkara *et al.* 2011) has found that there is a strong correlation between the number of retrieved oocytes and the live-birth rate in the fresh embryo transfer cycle: when the number of retrieved oocytes is 15, the number of retrieved oocytes has a positive correlation with the live birth rate; when the number of retrieved oocytes is between 10 and 20, the live birth rate tends to be stable, and when the number of retrieved oocytes is greater than 20, the live birth rate decreases with the increase of the number of retrieved oocytes. However, as the number of retrieved oocytes increases, the risk of ovarian hyperstimulation syndrome (OHSS) in the fresh embryo transfer cycle may increase (D'Angelo & Amso 2007). Some studies have also shown that the recruitment of a large number of oocytes during COS may lead to an increase in the proportion of immature oocytes and the aneuploidy rate, thus reducing the live birth rate (Haaf *et al.* 2009). However, to date, no studies have examined the relationship between the number of retrieved oocytes and the cumulative live birth rate in frozen-thawed embryo transfer cycles.

Female age is another important factor affecting the live birth rates. In 2006–2007, the predicted LBR for women with 15 oocytes retrieved in the age groups of 18–34, 35–37, 38–39, and 40 and older was 40, 36, 27, and 16%, respectively. Studies (Garrido *et al.* 2011) have found that age was a disadvantage for live birth rate for women over 35, with live birth rate and infant mortality rates falling rapidly for women over 40. Previous studies have shown that cumulative live birth rate (CLBR) increases with the number of oocytes retrieved, but this effect varies across age groups and plateaus beyond certain thresholds. When the age was between 30 and 34 years, there were three peaks in the number of eggs retrieved (15–18, 30, and >45). The overall trend was that CLBR increased with the increase of the number of eggs retrieved. When aged 35–39 years, CLBR was lower than in the three groups mentioned earlier, but the general trend was that CLBR increased with the number of eggs retrieved. When the age was ≥40 years, the CLBR reached the highest point when the number of eggs retrieved was 15–20, which was the same as before (Nie *et al.* 2024). This is consistent with our results. How to obtain appropriate oocytes according to the individual situation of the patient and reduce the incidence of OHSS is crucial. In addition, because the CLBR per egg retrieval cycle is closely related to the number of eggs retrieved, even if the current transfer cycle fails, the patient can still proceed to the next transfer cycle as long as the embryos are still available. Not only can ovarian stimulation and oocyte retrieval be avoided again, but also the cost can be reduced. If the patient does not have a live birth in this cycle, we can also accurately calculate the recommended number of oocytes obtained next time according to the model. For example, when the age is 34 years, the number of oocytes obtained is 10, the number of mature eggs is 6, and the number of high-scoring blastocysts is 4, the patient’s probability of live birth is about 60%, and no live birth is achieved. According to the prediction model, we recommend that the number of eggs retrieved in the next cycle be approximately 15, with a probability of live birth of more than 90%. However, IVF egg retrieval surgery is charged per time, and the cost of egg retrieval is not directly related to the number of eggs, and the cost of ten and one is the same. At present, the cost of IVF oocyte retrieval in regular hospitals in China is about 7,200–7,800 yuan (mainly including egg collection, egg pick-up, prokaryotic observation, and cleavage stage embryo culture). According to the different needle of oocyte retrieval and whether anesthesia is used, the charge is slightly different, but usually not more than 10,000 yuan. As all the patients enrolled in this experiment underwent ICSI after bilateral egg retrieval, we believe that the fewer the number of eggs retrieved, the higher the economic benefit, and the less the pain the patients have to bear.

In addition to egg-related indicators, hormone levels and ovarian stimulation protocols play a vital role in influencing IVF/ICSI outcomes. Studies have shown that elevated baseline follicle-stimulating hormone (FSH) levels are associated with diminished ovarian reserve and reduced cumulative live birth rates. Furthermore, the choice of COS protocol, including antagonist, microdose flare, or long luteal phase protocols, may impact both the quantity and quality of retrieved oocytes. For example, Alper & Fauser (2017) highlighted that different COS protocols lead to variation in live birth outcomes even in similar patient populations. Although our study accounted for ovulation stimulation protocols and hormone levels during data collection, these variables were not retained in the final predictive model due to limited statistical power and protocol heterogeneity. In future research, incorporating comprehensive hormonal profiles such as AMH and estradiol, as well as detailed COS strategies, would enhance the model’s explanatory power and provide a clearer comparison between egg retrieval and other influencing factors (Broekmans *et al.* 2006).

In recent years, clinical prediction models have been widely used in the diagnosis, treatment, and prognosis of diseases. A clinical prediction model, also known as a risk prediction model, is a model that predicts the outcome through statistics by including multiple variables (such as clinical indicators, biochemical indicators, and imaging). By building a clinical prediction model, patients’ clinical conditions can be predicted, such as disease occurrence, severity grading, risk, and outcome, helping doctors to more accurately assess patients’ disease risk and prognosis, and improving the accuracy and individualization of clinical decision-making (Moons *et al.* 2009, Steyerberg *et al.* 2013). At present, clinical indicators and laboratory indicators of patients have been used as predictors of live birth rate (Sunkara *et al.* 2011), but this study did not further screen the predictors of the live birth rate and analyzed the predictors of the cumulative live birth rate of IVF/ICSI for female patients of different ages by constructing a clinical prediction model. We found that the number of MII oocytes and the number of high-score blastocysts were effective predictors of cumulative live birth rate in women <35 years of age. The number of MII oocytes and the number of follicles under ultrasound were the most effective predictors of cumulative live birth rate in women 35 ≤ age <40 years. When the female age was >40 years, the effective predictor of cumulative live birth rate was only the number of retrieved oocytes. The number of retrieved oocytes becomes the best predictor of cumulative live birth with age, which may be related to the decline in ovarian reserve function and oocyte quality with age. For reference, a large retrospective cohort study by Asa (Magnusson *et al.* 2018) examined the relationship between the number of eggs required by patients to obtain the maximum CLBR and safety. They found that the live birth rate in fresh embryo transfer cycles increased with the number of eggs retrieved, but the incidence of severe OHSS increased with the number of eggs retrieved. It is particularly significant when it exceeds 18. In 2014, (Steward *et al.* 2014) Steward *et al.* analyzed the number of oocytes retrieved and pregnancy outcomes of 256,381 IVF/ICSI cycles in the United States from 2008 to 2010. The patients were divided into four groups according to the number of oocytes retrieved (0–5, 6–10, 11–15, 6–20, 21–25, >25). The live birth rate and the incidence of OHSS (ovarian hyperstimulation syndrome) were analyzed. The results showed that the pregnancy rate increased gradually (17, 31.7, and 39.3%) in the three groups with the number of oocytes obtained (0–5, 6–10, 11–15). When the number of oocytes obtained was more than 15, the live birth rate decreased gradually with the increase of the number of oocytes obtained. The incidence of OHSS gradually increased with the increase in the number of oocytes retrieved. In conclusion, when the number of oocytes retrieved is between 15 and 18, the incidence of OHSS can be controlled at an appropriate level. Our model only provides a reference for patients of different ages. In clinical practice, a more appropriate oocyte retrieval protocol should be formulated based on the experience of clinicians and the specific conditions of patients.

Although our model predicts that if 14 eggs are obtained, the live birth rate is only 50%, some studies have shown that the CLBR of the age group greater than 40 years is 19.00% (Nie *et al.* 2024). In order to deal with the effect of age on the number and quality of eggs, doctors often recommend that older women take some additional measures to improve their physical health and improve the quality of eggs. This includes regular and comprehensive physical examinations, dietary adjustments, and increased exercise. It is crucial for older women to obtain an adequate number of eggs during IVF. Despite the set of challenges associated with advancing age, older women still have the opportunity to fulfill their reproductive aspirations with physician guidance and reasonable efforts. It has been shown in the literature (Jia-Yin 2019) that the fertility of women over 40 years old gradually decreases with age. ART still has certain value for women aged 40–43 years, especially for those with a certain ovarian reserve (AMH >1.0 ng/mL). However, ART is not recommended for women over 44 years, and egg donation or adoption is strongly recommended for women over 46 years with ovarian failure. Specifically, we should consider the patient’s wishes and economic conditions comprehensively.

The ‘cumulative live-birth rate’ in this report is the transfer cycle of all embryos formed after a single oocyte retrieval cycle through the end of embryo transfer and could include multiple pregnancies and twins, but it was assigned to be 1 in our models as long as a patient had a live birth, with reference to dichotomous variables, and 0 otherwise.

Since the number of MII oocytes, the number of high-score blastocysts, and the number of follicles under ultrasound are not good guides for clinical follicle recruitment, we want to use the number of retrieved oocytes to replace these predictors, so as to guide the COS protocol, drug type, and dose during follicle recruitment. Moreover, we found a linear regression relationship between the number of retrieved oocytes and the number of MII oocytes, the number of high-score blastocysts, and the number of follicles under ultrasound, thus making our idea possible. Through the analysis, we found that when the female age <35 years, when the number of retrieved oocytes is 10, 15, and 20, the probability of live birth is 50%+, 99, and 99.9%, respectively. When the female age was 35 years and less than 40 years, when the number of eggs obtained was 15, 20, and 25, the probability of live birth was 60%–70, 90, and 95%+, respectively. And when the female was >40 years, when the number of retrieved oocytes was 14, the probability of live birth was 50%. The results of this study suggest that clinicians are recommended to make a reasonable COS protocol according to the age of the female patient, so as to achieve the best live birth and reduce the risk of OHSS.

## Conclusion

Through the construction of a clinical prediction model, we found the predictors of cumulative live birth rate for infertile women of different ages who underwent IVF/ICSI treatment. When the age was <35 years, the predictors of cumulative live birth rate were the number of MII oocytes and the number of high-score blastocysts; when 35 ≤ age <40 years, the predictors of cumulative live birth rate were the number of MII oocytes and the number of follicles under ultrasound; when the age was >40 years, the predictor of cumulative live birth rate was the number of retrieved oocytes. Combined with the linear regression equation, the probability of live birth was 50%+, 99, and 99.9% in females <35 years old when the number of retrieved oocytes was 10, 15, and 20, and 60%–70, 90 and 95%+ in females 35 ≤ age <40 years when the number of retrieved oocytes was 15, 20, and 25, respectively. In women aged 40 years or older, the probability of live birth is 50% when the number of retrieved oocytes is 14.

## Declaration of interest

The authors decalare that there are no conflicts of interest that can be perceived to prejudice the impartiality of the reserach reported here.

## Funding

This research was funded by the National Key R&D Program of Chinahttps://doi.org/10.13039/501100012166, grant number 2024YFC2707200; the National Natural Science Foundation of Chinahttps://doi.org/10.13039/501100001809, grant number 82001635; the Hefei Comprehensive National Science Center Medical-Industrial Integration Medical Equipment Innovation Research Platform Project, grant number 4801001202; the Clinical Medical Research Transformation Project of Anhui Province, grant number 202204295107020012; the Foundation for Selected Scientists Studying Abroad of Anhui Province, grant number 2022LCX015; and the Innovation and Entrepreneurship Training Program for Undergraduate Students of Anhui Medical University, grant numbers 202310366015 and S202310366009.

## Author contribution statement

CY contributed to formal analysis, methodology and writing the original draft. PC helped in data curation, writing, review and editing. HL contributed to the conception and design of the study. ML helped with the data analysis. XJ was responsible for conceptualization. LX helped collect the data. DJ contributed to the acquisition of data. ZZ critically revised the manuscript for important intellectual content. YC critically revised the manuscript. WZ helped draft the article and critically revised the manuscript. All authors approved the final manuscript.

## Data availability

Data are not publicly available due to containing information that could compromise the privacy of research participants.
